# Effect of the black chokeberry (*Aronia melanocarpa* (Michx.) Elliott) juice acquisition method on the content of polyphenols and antioxidant activity

**DOI:** 10.1371/journal.pone.0219585

**Published:** 2019-07-18

**Authors:** Zbigniew Kobus, Rafał Nadulski, Kamil Wilczyński, Marta Kozak, Tomasz Guz, Leszek Rydzak

**Affiliations:** Department of Food Engineering and Machines, University of Life Sciences, Lublin, Poland; Institute for Biological Research, SERBIA

## Abstract

The primary objective of the study was to determine the effect of process conditions on extraction efficiency and the total amount of released polyphenols and antioxidant activity (AA) in black chokeberry juice. The study samples were fruits of black chokeberry (*Aronia melanocarpa* (Michx.) Elliott) cv. Galicjanka. In the study, two kinds of presses—piston press and twin gear juice extractor—were used, and two raw material pretreatment methods—freezing and thawing and enzymatic liquefaction—were applied. The study showed that pressing efficiency depends on the design of press and the nature of pretreatment. The highest pressing efficiency was obtained using the twin gear juice extractor. Enzymatic liquefaction of shredded fruits significantly increased the efficiency of pressing by the piston press. The type of press and the pretreatment method used had an effect on the quality traits of the extracted juices. The highest content of soluble solids was obtained for fruits not subjected to any pretreatment and pressed using the twin gear press. The highest total phenolic content was obtained in juice extracted using the piston press from shredded fruits subjected to enzymatic treatment at 45°C. A higher total phenolic content was also a characteristic of juice obtained from fruits not subjected to any pretreatment and extracted using the twin gear press. The capacity of the black chokeberry juices for free radical quenching oscillated around the level of approximately 90%. The study showed that the application of suitable processing methods is necessary for the acquisition of products with desirable quality traits.

## Introduction

*Aronia melanocarpa* (Michx.) Elliott, called the black chokeberry, is a species of shrubs in the rose family. This plant is widely grown in Europe, including Poland. Black chokeberry (*Aronia melanocarpa* (Michx.) Elliott) fruits are a valuable material for the production of juices because of its high content of biologically active components [1]. Black chokeberry fruits are characterized by high content of vitamin C and polyphenolic compounds, especially anthocyanins [2]. Polyphenols are probably the most important components of black chokeberry fruits and are responsible for most of their beneficial health properties. These compounds exhibit strong antioxidant properties and can reduce the risk of occurrence of certain lifestyle diseases and inhibit aging processes [3, 4, 5]. Black chokeberry fruits contains approximately 0.3% of tannins, the composition of which includes catechins and their dimers, quercetin, and tannins responsible for the pungent, slightly bitter taste of the fruits. Because of its characteristic taste, fresh black chokeberry is rarely consumed, and consumers most often prefer its processed forms—jams or juices. Tannins also have a stabilizing effect on anthocyanin pigments responsible for the dark violet color of the fruits. These pigments impart the specific color to the juice of aronia fruits, with various hues depending on its acidity [4, 6].

Berries, including aronia fruits, can be used for the production of functional foods, provided that their bioactive components are not lost during processing [6–9]. On the one hand, one of the most important factors in the process of juice extraction on an industrial scale is the achievement of the highest possible extraction efficiency. On the other hand, for the consumer, the most important quality indicators of the purchased juice are the health-promoting properties and sensory features. Therefore, the technological conditions of the production process should be chosen in order to meet both these conditions at the same time. Research has shown that black chokeberry fruits contain approximately 0.63–0.75% of pectins, including pectin fractions, which cause high pulp viscosity, because of which the fruits are classified as materials that are hardly susceptible to pressing [10, 11]. The application of thermal or enzymatic treatment to fruits can facilitate the process of pressing and improve extraction efficiency. Treatment of this kind is also favorable because of the possibility of acquiring juice with a high content of polyphenols, including anthocyanins, which are found mainly in the skin of aronia fruits. In cells, these compounds occur in vacuoles in the form of granules of varying sizes. Cell walls do not contain anthocyanins and are composed of a compact network of cellulose and hemicelluloses embedded in a pectin matrix, Therefore, the extraction of anthocyanins from cell structures during conventional pressing is difficult and less efficient than that of other phenolic compounds. In addition, it should be noted that after pressing, a significant amount of anthocyanins remains in the pomace [12, 13, 14].

For the processing of pulp of fruits (black currant, black chocoberry), it is recommended to apply enzymatic maceration, thermal treatment, or combined treatment, which can transform the structure-forming components and largely determine the efficiency of the process of pressing. As shown by several studies, treatment of this type not only improves the efficiency of extraction but also contributes to an increase in the degree of release of polyphenols and in free-radical quenching activity [12, 13, 15, 16]. In recent years, alternative methods have been investigated to treat fruits and vegetables prior to pressing, for example, by freezing and thawing of pulp [17, 18]. Freezing is considered to be an almost noninvasive method of food preservation that does not cause degradation of biologically active components.

The process of fruit juice production should be highly efficient, which allows the acquisition of juices with a high content of soluble solids, low pH, and a high content of health-promoting components such as polyphenols, carotenoids, vitamins, macroelements, and microelements.

Therefore, the primary objective of the present study was to determine the effect of process conditions on extraction efficiency, total amount of released polyphenols, and antioxidant activity (AA). The scope of the study also included the determination of juice quality parameters such as the content of soluble solids, pH, and density. Within the framework of the experiment, fruits were pressed using the following two types of presses with different designs: piston press and twin gear juice extractor. Two raw material pretreatment methods were applied—freezing and thawing and enzymatic liquefaction. The originality of the study is based on the analysis of the impact of design solutions for presses and pulp processing methods on the quality of the obtained juice. The novelty of our work lies in the use of freezing and thawing of the pulp as a new form of pretreatment for juice obtained by pressing raw materials of plant origin.

## Materials and methods

The study samples were fruits of black chokeberry (*Aronia melanocarpa* (Michx.) Elliott) cv. Galicjanka harvested in 2016. Black chokeberry was cultivated, harvested, and supplied by a specialist horticulture farm in Samoklęski Kolonia Druga (Poland). Fruits accepted for the tests were characterized according to technological ripeness parameters. The tests were conducted on healthy fruits without any mechanical damage. Prior to testing, the fruits were washed, dried on a blotting paper, and then divided into portions of 300 g each.

### Experimental procedure

**Fig 1:**
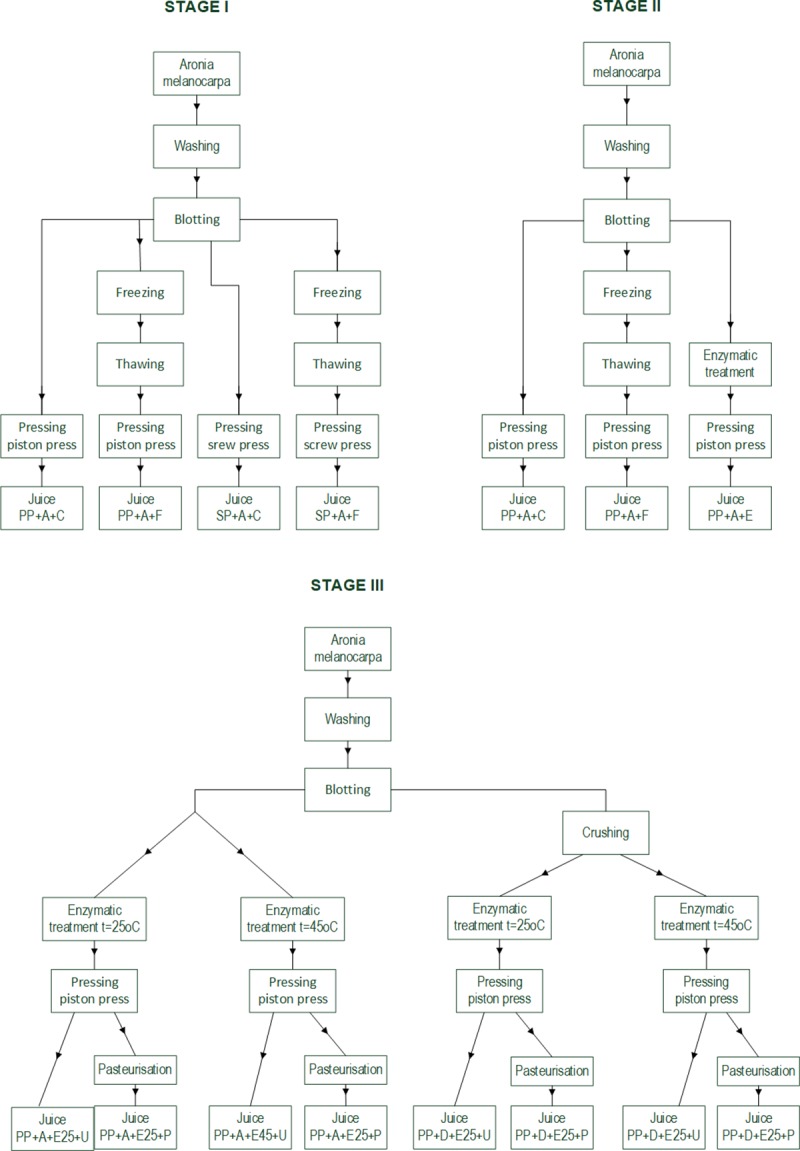
Scheme of the experiment. Abbreviations: SP—twin gear juice extractor; PP—cylindrical piston press; A—nonshredded fruits; D—shredded fruits; C—sample not subjected to any pretreatment (control); F—sample after pretreatment by freezing and thawing prior to pressing; E—sample after enzymatic pretreatment; E25—sample subjected to enzymatic treatment at 25°C; E45—sample subjected to enzymatic treatment at 45°C; U—nonpasteurized juice; P—pasteurized juice.

The experiment was conducted in three stages: in the first stage, the extraction efficiency and juice quality were compared between the twin gear juice extractor and the piston press, considering the pretreatment of fruits by freezing and thawing prior to the pressing (stage I). In the second stage, the effect of the pretreatment on extraction efficiency and quality of juice obtained using the piston press when pressing whole fruits was estimated (stage II). In the third stage, the effect of variants of enzymatic treatment of whole and shredded fruits on extraction efficiency and the quality of juice obtained using the piston press was analyzed (stage III). The experimental design is presented in Fig 1.

In the study, two types of preliminary treatment were applied prior to the extraction of juice from black chokeberry fruits. The first pretreatment included freezing and thawing of aronia fruits prior to juice extraction. The fruits were kept frozen at −20°C in a standard freezer (F6243W, Gorenje Group, Velenje, Slovenia) and then thawed for 24 h in an incubator (SUP-4, Wamed, Warsaw, Poland) at 20°C. The second pretreatment included enzymatic treatment of fruits prior to juice extraction. The enzymatic treatment was applied to both whole and shredded fruits. The fruits were treated with an enzymatic preparation (Pektoenzym, Biowin Sp. z o.o., Łódź, Poland) at a concentration of 0.2 ml/kg of raw material. During enzymatic liquefaction, the raw material was kept in an incubator (SUP-4, Wamed) at 25°C or 45°C for 6 h. Prior to the enzymatic treatment, the fruits were shredded using a crusher (Zelmer 491.4, Zelmer, Rzeszów, Poland). Next, juice obtained after the enzymatic liquefaction was heated at 90°C for 5 minutes to inactivate the enzymes contained in the enzymatic preparation used.

### Extraction process

Two types of presses were used for juice extraction—a piston press with a basket or the authors’ own design [18] and a twin gear juice extractor (Green Star Elite 3000, Tribest). For the basket press, 300 g portions of raw material (whole or shredded fruits) were placed in a bag; the bag was then placed in the working chamber and loaded using the piston. The piston was moved at a constant speed of 0.5 mm s^−1^. The extraction process was conducted until a pressure of 40 kN was achieved and then stopped. The extracted juice was collected in containers. For the twin gear press, 300 g portions of whole fruits were added through the charging funnel to the working chamber, where the fruits were first crushed and the juice was then extracted using two parallel worm gears. The rotation speed of the gears was 1.8 s^−1^. Finally, the extracted juice was collected in containers.

### Extraction efficiency

After every pressing, the mass of the acquired juice was measured. The extraction efficiency for both presses was determined using the following formula:

$$W=\left(M_{j}/M_{i}\right)\times 100\%$$

where *W* is the pressing efficiency (%), *M*_*j*_ is the mass of juice after pressing (kg), and *M*_*i*_ is the mass of input material (kg).

### Determination of soluble solids content, juice pH, and density

The soluble solids content in the juice was determined using a refractometric method [19] with ATAGO PAL-3 refractometer (ATAGO, Tokyo, Japan). The pH of juice was determined by the pH-metric method [20] with CP-411 pH meter (Elmetron, Zabrze, Poland). Juice density was determined by the pycnometric method at 20°C using distilled water as a model liquid.

### Total phenolic content (TPC)

The total phenolic content (TPC) of the extracted juices of aronia fruits was measured by the modified Singleton method based on the Folin-Ciocalteu reagent [21]. For this purpose, 2 ml of the Folin-Ciocalteu reagent was added to 0.2 ml of juice. After 3 min, the reaction environment was alkalized by adding 2 ml of a 6% solution of sodium carbonate. Next, after waiting for 3 min, distilled water was added to increase the total volume up to 25 ml. The solutions were kept for 30 min at room temperature in dark. Absorbance was determined using a UV–1800 spectrophotometer (Shimadzu, Japan) at a wavelength of λ = 760 nm. The TPC was expressed as an equivalent of mg of gallic acid in 100 ml of fresh juice.

### Antioxidant activity

The AA of the extracted juice was determined by the method of reduction of 2,2-diphenyl-1-picrylhydrazyl (DPPH) [22]. Approximately 5.8 ml of methanol solution containing DPPH radical with a concentration of 6 ×10^−5^ mol∙dm^−3^ was added to 0.2 ml of juice. The solutions were kept for 30 min at room temperature in dark. The decrease in absorbance was determined by the spectrophotometric method at the maximum absorbance of DPPH radical and at a wavelength of λ = 516 nm. The percentage content of residual (nonreduced) DPPH radical was calculated using the following equation:

$$\%\mathrm{inhibition}=\left(\left[A_{0}-A_{m}\right]/A_{0}\right)\times 100\%$$

where *A*_*0*_—absorbance of DPPH radical solution and *A*_*m*_—mean value of absorbance of juice solution with DPPH radical.

### Statistical analysis

Each measurement was performed in three replicates. Statistical analysis was performed with Statistica [23] using mono- and multifactorial analysis of variance. The significance of differences was determined using Tukey’s LSD test at a significance level of p<0.05. The results of the experiments are shown in Tables and in a graphical form in graphs. The graphs show mean values and whiskers representing standard deviations, while the Tables show the mean values and standard deviations.

## Results and discussion

The statistical analysis of the results revealed a significant (p<0.05) effect of the type of press and the pretreatment method on the efficiency of the pressing process and the quality of the extracted juices. The health-promoting value of the juices was strongly correlated with the pressing process conditions.

### Extraction efficiency

**Fig 2:**
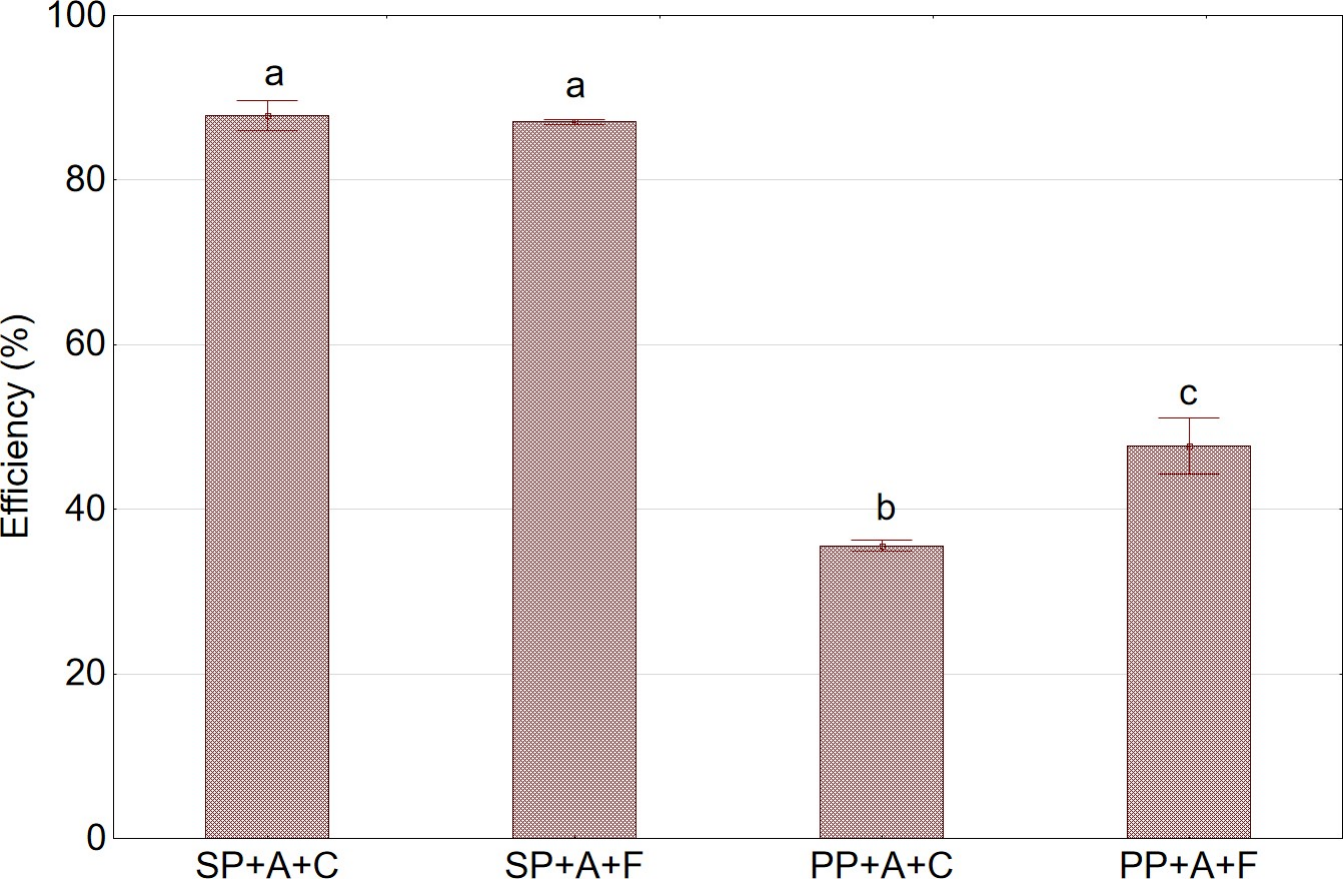
Pressing efficiency in relation to press type and pretreatment method. Abbreviations: SP—twin gear juice extractor; PP—cylindrical piston press; A—nonshredded fruits; C—sample not subjected to any pretreatment (control); F—sample after pretreatment by freezing and thawing prior to pressing. ^a,b,c^Values in a column marked with the same letter are not significantly different (p>0.05).

The study demonstrated that pressing efficiency depends on the type of press used and the nature of the pretreatment of raw material prior to pressing. The efficiency of the pressing of fruits not subjected to pretreatment using the twin gear extractor was nearly 150%, which was higher than that obtained with the piston press (Fig 2). This obtained efficiency was related to the design of the twin gear press, as in the working assembly, the pressing is preceded by preliminary crushing of raw material by two grooved cylindrical crushers integrated with a pair of pressing worm gears. The application of pretreatment consisting of fruit freezing and thawing before pressing did not cause any increase in pressing efficiency for the twin gear press, whereas it caused a significant increase (34.1%, p<0.05) in the efficiency of pressing with basket-piston press. Slow freezing caused the formation of large ice crystals in cells and intercellular spaces [24, 25]. The growth of the crystals could have caused damage to the cells and considerable seepage of cellular juice after thawing [24, 26].

The efficiency of pressing with the twin gear juice extractor was still higher than that obtained with the piston press by more than 80%, despite the augmentation of the process by pretreatment consisting of fruit freezing and thawing prior to pressing. The use of the twin gear juice extractor allowed to achieve pressing efficiency of above 85%, which is equivalent to the efficiency achieved under industrial conditions using basket press after the application of enzymatic treatment.

**Fig 3:**
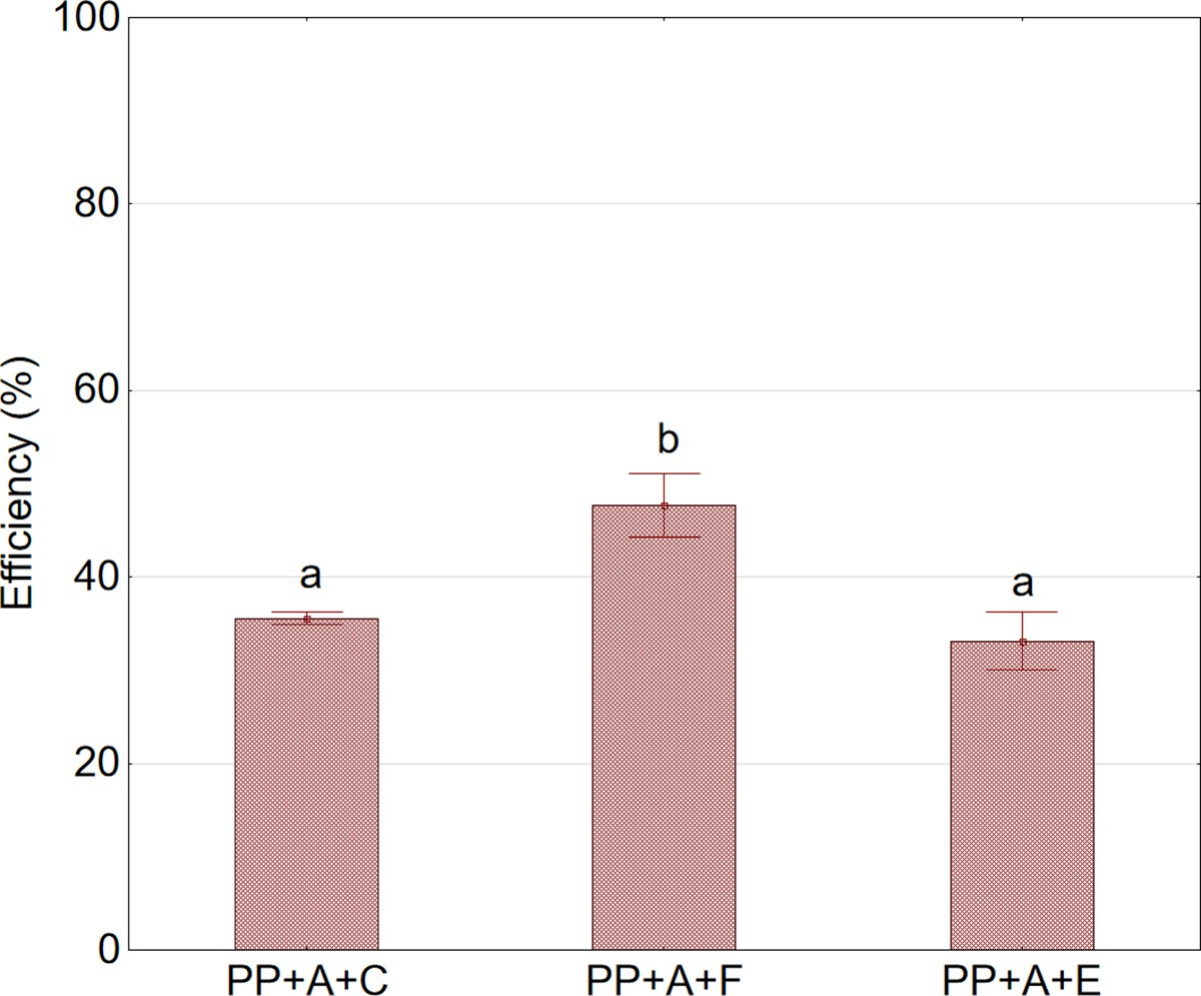
Efficiency of pressing using the piston press in relation to pretreatment prior to pressing. Abbreviations: SP—twin gear juice extractor; A—nonshredded fruits; D—shredded fruits; C—sample not subjected to any pretreatment (control); F—sample after pretreatment by freezing and thawing prior to pressing; E—sample after enzymatic pretreatment.^a,b^Values in a column marked with the same letter are not statistically significantly different (p>0.05).

Fig 3 shows the effect of pretreatment on the efficiency of whole aronia fruits pressed using the piston press. As can be seen from the graph, an increase in pressing efficiency by approximately 39%, relative to the control sample, was observed after the application of pretreatment consisting of fruit freezing and thawing prior to pressing.

**Fig 4:**
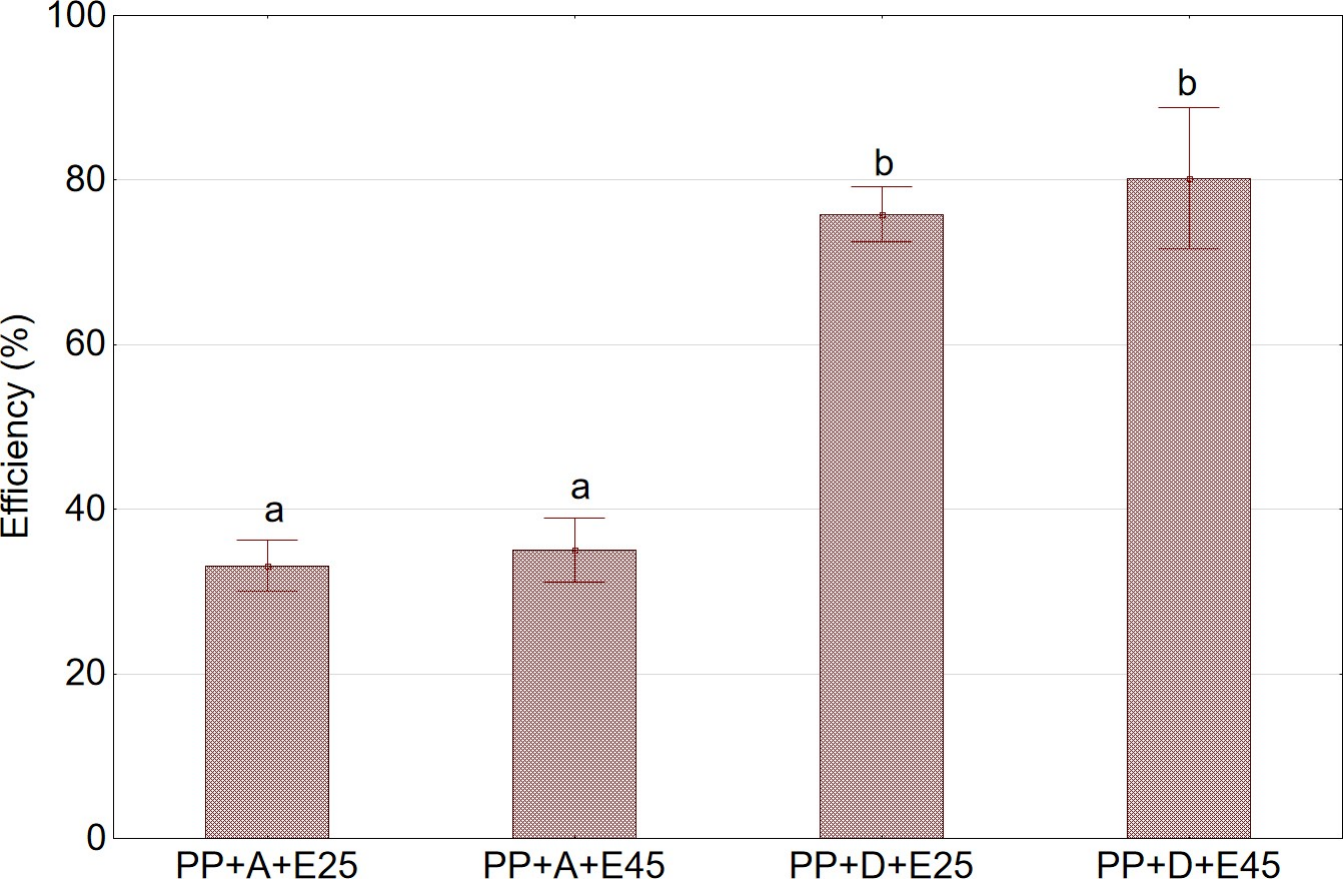
Efficiency of pressing using the piston press in relation to enzymatic treatment. Abbreviations: PP—cylindrical piston press; A—nonshredded fruits; D—shredded fruits; E25—sample subjected to enzymatic treatment at 25°C; E45—sample subjected to enzymatic treatment at 45 ^o^C. ^a,b^Values in a column marked with the same letter are not statistically significantly different (p>0.05).

In the next stage of the study, two variants of enzymatic treatment were analyzed with respect to an increase in the efficiency of pressing using the piston press. It was found that the use of the enzymatic preparation caused a significant increase in pressing efficiency for shredded fruits. Compared to whole fruits, the observed increase in pressing efficiency was nearly 130% (Fig 4). It should be noted that enzymatic treatment did not cause liquefaction of the skin of aronia fruits. In addition, increase in the temperature of enzymatic liquefaction of both the fruits and the pulp from 25°C to 45°C did not cause any significant (p>0.05) increase in the efficiency of the pressing process. The study showed that the application of enzymatic treatment prior to pressing allows to achieve efficiency at a level close to 80%, but still lower than that obtained with twin gear presses.

Another important issue related to the use of various types of presses and pretreatment methods is the quality of the juice obtained.

### Soluble solids content

The content of soluble solids in aronia juice depended on the method of extraction (Tables 1–3). It was affected by both the type of presses used and the nature of pretreatment.

**Table 1:** Soluble solids content (^o^Bx) in acquired juices in relation to press type and the pretreatment method.

Sample code	Soluble solids content (^o^Bx)
SP+A+C	21.37±0.06^a^
SP+A+F	18.03±0.06^b^
PP+A+C	20.67±0.06^c^
PP+A+F	17.73±0.06^d^

^a,b,c,d^Values in a column marked with the same letter are not statistically significantly different (p>0.05).

Abbreviations: SP—twin gear juice extractor; PP—cylindrical piston press; A—nonshredded fruits; C—sample not subjected to any pretreatment (control); F—sample after pretreatment by freezing and thawing prior to pressing.

**Table 2:** Soluble solids content (^o^Bx) in juice acquired using the piston press in relation to the type of pretreatment.

Sample code	Soluble solids content (^o^Bx)
PP+A+C	20.67±0.06^a^
PP+A+F	17.73±0.06^b^
PP+A+E	19.50±0.10^c^

^a,b,c^Values in a column marked with the same letter are not statistically significantly different (p>0.05).

Abbreviations: PP—cylindrical piston press; A—nonshredded fruits; C—sample not subjected to any pretreatment (control); F—sample after pretreatment by freezing and thawing prior to pressing; E—sample after enzymatic pretreatment.

**Table 3:** Soluble solids content (^o^Bx) in juice acquired using the piston press in relation to the type of enzymatic treatment.

Sample code	Soluble solids content (^o^Bx)
PP+A+E25+U	19.50±0.10^a^
PP+A+E25+P	19.50±0.10^a^
PP+A+E45+U	19.53±0.06^a^
PP+A+E45+P	19.47±0.06^a^
PP+D+E25+U	20.57±0.06^b^^,^^c^
PP+D+E25+P	20.37±0.06^b^
PP+D+E45+U	21.03±0.06^d^
PP+D+E45+P	20.70±0.10^c^

^a,b,c,d^Values in a column marked with the same letter are not statistically significantly different (p>0.05).

Abbreviations: PP—cylindrical piston press; A—nonshredded fruits; D—shredded fruits; E25—sample subjected to enzymatic treatment at 25°C; E45—sample subjected to enzymatic treatment at 45 ^o^C; U—nonpasteurized juice; P—pasteurized juice.

The content of soluble solids in juice obtained using the twin gear press was higher than that using basket-piston press. The application of pretreatment in the form of fruit freezing and thawing prior to pressing caused, irrespective of the type of press used, a decrease in soluble solids content in juice by approximately 14–16%. This may be due to the freezing water contained in cellular juice and its subsequent migration to the juice obtained as a result of pressing. Stewart [27] observed an increase in the soluble solids content in the juice produced from frozen berries. For juice obtained from whole fruits by pressing with the basket-piston press, it was found that the application of enzymatic treatment caused a decrease in soluble solids content in the juice by approximately 6%, while fruit freezing and thawing prior to pressing caused a decrease by approximately 16% relative to the control sample. After the application of enzymatic treatment to shredded fruits, the soluble solids content in extracted juice was 20.57–21.03^o^Bx, and it was similar to that in the control sample (20.67^o^Bx). The application of enzymatic treatment of pulp at a higher temperature caused this slight increase in the soluble solids content in juice. After pasteurization of juice from shredded fruits, a small decrease in the soluble solids content was observed in juice obtained after enzymatic liquefaction at both 25°C and 45°C. In addition, pasteurization of juice caused the deactivation of enzymes. As reported in the literature [28], the presence of enzymatic preparations in juice causes more effective degradation of polysaccharides contained in shredded fruits, and thus may cause a higher soluble solids content in juice.

### Juice pH

Acidity is one of the principal juice quality indicators, and therefore, in juice production, it is desirable to achieve a low level of juice pH. In the present study, the method of juice acquisition had only a slight effect on juice pH. For the control sample, the juice obtained with the twin gear extractor had lower acidity than that obtained with the piston press. Furthermore, pretreatment consisting of fruit freezing and thawing caused a slight increase in juice pH relative to the control sample (Tables 4 and 5).

**Table 4:** pH of acquired juices in relation to press type and the pretreatment method. ^a,b,c^Values in a column marked with the same letter are not statistically significantly different (p>0.05).

Sample code	pH
SP+A+C	3.307±0.015^a^
SP+A+F	3.327±0.006^a,c^
PP+A+C	3.213±0.006^b^
PP+A+F	3.353±0.015^c^

Abbreviations: SP—twin gear juice extractor; PP—cylindrical piston press; A—nonshredded fruits; C—sample not subjected to any pretreatment (control); F—sample after pretreatment by freezing and thawing prior to pressing.

**Table 5:** pH of juices acquired using the piston press in relation to the type of pretreatment.

Sample code	pH
PP+A+C	3.213±0.006^a^
PP+A+F	3.353±0.015^b^
PP+A+E	3.250±0.010^c^

^a,b,c^Values in a column marked with the same letter are not statistically significantly different (p>0.05).

Abbreviations: PP—cylindrical piston press; A—nonshredded fruits; C—sample not subjected to any pretreatment (control); F—sample after pretreatment by freezing and thawing prior to pressing; E—sample after enzymatic pretreatment.

Juice obtained after enzymatic liquefaction was characterized by a different level of pH (Table 6). Thermal treatment of juices obtained from the whole and shredded fruits after enzymatic treatment at 25 ^o^C and 45 ^o^C did not cause any change in their acidity.

**Table 6:** pH of juices acquired using the piston press in relation to the type of enzymatic treatment.

Sample code	pH
PP+A+E25+U	3.250±0.010^a^
PP+A+E25+P	3.257±0.015^a^
PP+A+E45+U	3.293±0.006^b^
PP+A+E45+P	3.283±0.006^b^
PP+D+E25+U	3.150±0.010^c^
PP+D+E25+P	3.143±0.006^c^
PP+D+E45+U	3.190±0.010^d^
PP+D+E45+P	3.203±0.006^d^

^a,b,c,d^Values in a column marked with the same letter are not statistically significantly different (p>0.05).

Abbreviations: PP—cylindrical piston press; A—nonshredded fruits; D—shredded fruits; E25—sample subjected to enzymatic treatment at 25°C; E45—sample subjected to enzymatic treatment at 45°C; U—nonpasteurized juice; P—pasteurized juice.

### Juice density

Crude juice obtained directly after pressing contains mechanical suspensions formed from parts of tissues and cells. The density of the analyzed juices were similar. Statistically significant (p<0.05) differences in density were observed between juices pressed by the twin gear press and the basket-piston press (Table 7). The density of juice obtained with the twin gear press was higher than that with the basket-piston press, irrespective of the pretreatment applied. However, after pretreatment consisting of fruit freezing and thawing, the density of juice decreased slightly relative to the control sample (Tables 7 and 8).

**Table 7:** Density (kg·m^−3^) of acquired juices in relation to press type and the pretreatment method.

Sample code	Density (kg·m^−3^)
SP+A+C	1.086±0.002^a^
SP+A+F	1.072±0.002^b^
PP+A+C	1.077±0.002^b^
PP+A+F	1.063±0.002^c^

^a,b,c^Values in a column marked with the same letter are not statistically significantly different (p>0.05).

Abbreviations: SP—twin gear juice extractor; PP—cylindrical piston press; A—nonshredded fruits; C—sample not subjected to any pretreatment (control); F—sample after pretreatment by freezing and thawing prior to pressing.

**Table 8:** Density (kg·m^−3^) of juices acquired using the piston press in relation to type of pretreatment.

Sample code	Density (kg·m^−3^)
PP+A+C	1.077±0.002^a^
PP+A+F	1.063±0.002^b^
PP+A+E	1.073±0.003^a^

^a,b,c^Values in a column marked with the same letter are not statistically significantly different (p>0.05).

Abbreviations: PP—cylindrical piston press; A—nonshredded fruits; C—sample not subjected to any pretreatment (control); F—sample after pretreatment by freezing and thawing prior to pressing; E—sample after enzymatic pretreatment.

There were no statistically significant differences in the juice density obtained after enzymatic treatment of whole and shredded fruits (Table 9).

**Table 9:** Density (kg·m^−3^) of juices acquired using the piston press in relation to the type of enzymatic treatment.

Sample code	Density (kg m^−3^)
PP+A+E25+U	1.073±0.003^a^
PP+A+E25+P	1.072±0.003^a^
PP+A+E45+U	1.073±0.003^a^
PP+A+E45+P	1.071±0.004^a^
PP+D+E25+U	1.078±0.003^a^
PP+D+E25+P	1.078±0.002^a^
PP+D+E45+U	1.078±0.003^a^
PP+D+E45+P	1.077±0.003^a^

^a,b,c^Values in a column marked with the same letter are not statistically significantly different (p>0.05).

Abbreviations: PP—cylindrical piston press; A—nonshredded fruits; D—shredded fruits; E25—sample subjected to enzymatic treatment at 25°C; E45—sample subjected to enzymatic treatment at 45 ^o^C; U—nonpasteurized juice; P—pasteurized juice.

### Total phenolic content

The content of polyphenols is an important parameter that affects the quality of fruit juices. Aronia fruits are rich in phenolic compounds, and their content depends on the fruit variety, attaining levels as high as 3,000 mg GEA/100 g FW [29]. Products derived from aronia, mainly juices, are characterized by TPC significantly lower than expected; this is because during the processing of juices by the traditional pressing process, most of the phenolic compounds present in the fruits are retained in the pomace due to noncovalent bonds within the cell wall [30]. In the present study, the content of polyphenols was determined at levels from 281.5 to 558.0 mg GAE/100 ml of juice. These values are in agreement with the results reported by other studies [29, 31]. Wangensteen et al. [29] analyzed aronia juices and obtained TPC in the range of 675 to 750 mg GAE/100 ml of juice, while Bijak et al. [31] obtained approximately 480 mg GAE/100 ml of juice.

**Fig 5:**
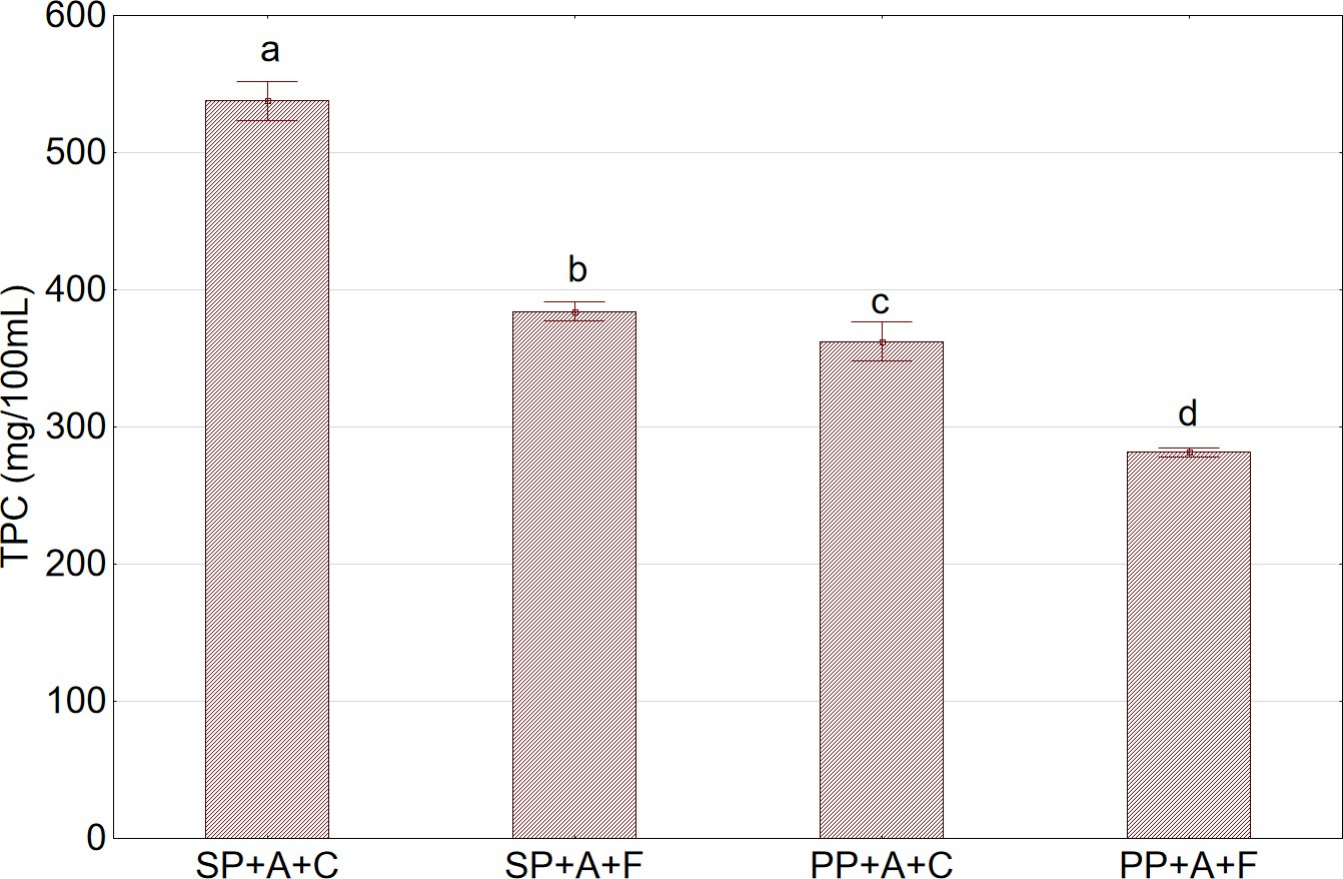
Total phenolic content (TPC) in juice in relation to press type and the method of pretreatment prior to pressing. Abbreviations: SP—twin gear juice extractor; PP—cylindrical piston press; A—nonshredded fruits; C—sample not subjected to any pretreatment (control); F—sample after pretreatment by freezing and thawing prior to pressing. ^a,b,c^Values in a column marked with the same letter are not statistically significantly different (p>0.05).

Furthermore, statistical analysis revealed that the TPC in aronia juice depended on the type of press used and the pretreatment of raw material prior to pressing. The TPC in juices acquired using the twin gear extractor was higher than that in juices obtained from the basket press (Fig 5). The differences were probably caused by the migration of a greater number of solid particles to juice extracted using the twin gear press as compared to that in juice produced using the piston press. Phenolic substances contained in fruits occur mainly in the fruit skin and its immediate vicinity; thus, the higher concentration of solid substances in the juice, mainly from the skins may be reflected in a higher content of phenolic substances. This fact was also confirmed by the higher density of juices extracted using the twin gear press.

The application of pretreatment consisting of fruit freezing and thawing prior to pressing had an unfavorable effect on the content of polyphenols in aronia juices. For the twin gear press, it caused a decrease in the TPC by approximately 28% and for the basket press by approximately 22%. Literature data on the effect of the process of freezing on the content of polyphenols in fruit juices are not well understood. Piłat and Zadernowski [32] observed a positive effect of the freezing on the content of polyphenols in the juice of lingonberry fruits. They also demonstrated that fruit freezing prior to pressing allows the extraction of approximately 60% of polyphenols contained in lingonberry fruits, while juice obtained by pressing fruits stored in a refrigerator at a temperature of approximately 4°C allows the extraction of these compounds at a level of approximately 50%. However, Mirsaeedghazi et al. [33] stored frozen pomegranate juice at approximately −25°C and observed a decrease in the TPC with an extension in the time of storage. The end effect of freezing on the content of polyphenols in fruit juices is a resultant of two factors with opposite effects. On the one hand, freezing and thawing cause damage to cell walls and facilitate the release of additional amounts of polyphenols in the juice during the process of pressing. On the other hand, the long-lasting process of thawing of fruits exposed to the effect of oxygen causes partial oxidation of polyphenolic compounds.

**Fig 6:**
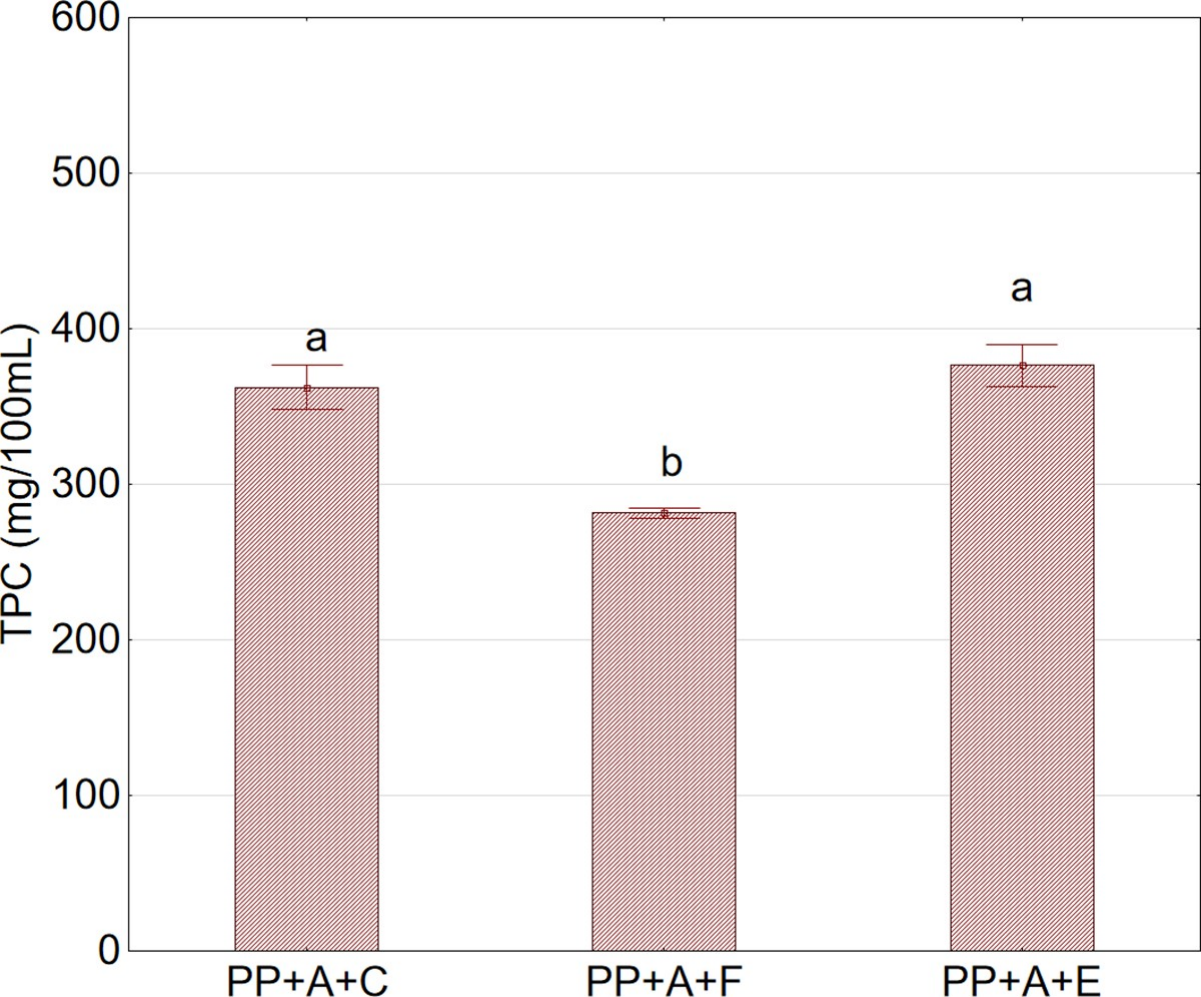
Total phenolic content (TPC) in juice extracted using the piston press in relation to the method of pretreatment prior to pressing. Abbreviations: PP—cylindrical piston press; A—nonshredded fruits; C—sample not subjected to any pretreatment (control); F—sample after pretreatment by freezing and thawing prior to pressing; E—sample after enzymatic pretreatment. ^a,b^Values in a column marked with the same letter are not statistically significantly different (p>0.05).

**Fig 7:**
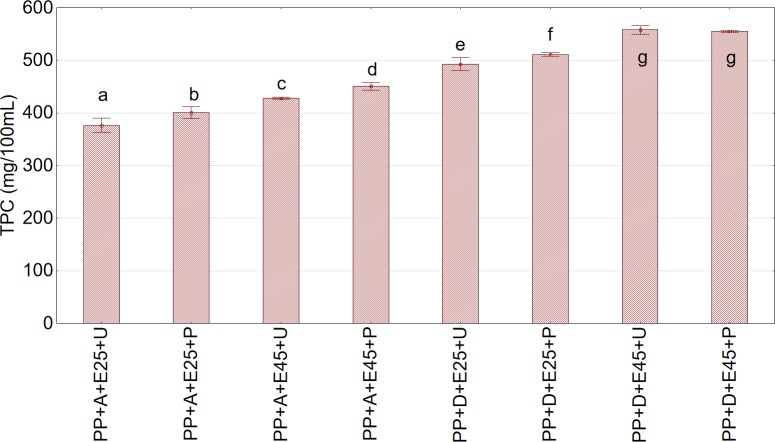
Total phenolic content (TPC) in pasteurized and nonpasteurized juice extracted using the piston press from fruits subjected to enzymatic treatment. Abbreviations: PP—cylindrical piston press; A—nonshredded fruits; D—shredded fruits; E25—sample subjected to enzymatic treatment at 25°C; E45—sample subjected to enzymatic treatment at 45 ^o^C; U—non-pasteurized juice; P—pasteurized juice. ^a,b,c^Values in a column marked with the same letter are not statistically significantly different (p>0.05).

The application of enzymatic treatment had a significant positive effect on TPC in the juices. The content of polyphenols was significantly related to fruit shredding, temperature of enzymatic treatment, and thermal treatment consisting of pasteurization of the juices (Figs 6 and 7). The enzymatic treatment of shredded fruits resulted in a higher content of polyphenols in the juices than that of whole aronia fruits. The higher temperature of enzymatic treatment (45°C) of pulp also caused an increase in the TPC in the juices. The observed relationships can be explained by more effective action of the enzymatic preparation on the cell structures of shredded fruits and the higher activity of the preparation at the higher temperature. The results regarding the positive effect of enzymatic treatment on increased content of polyphenols in juices are in agreement with the results obtained by other studies [34, 35, 36, 37].

Under the experimental conditions, a higher TPC was observed in the juices after the process of pasteurization. This increase, however, should be considered highly debatable. It may be caused by the imperfect method of determination of TPC, consisting of recording spectra of color complexes that are formed by phenolic compounds with the Folin reagent. Probably, at an elevated temperature (i.e., the temperature at which pasteurization is performed), additional groups of color complexes are formed that could falsify the actual content of polyphenols in juices.

### Antioxidant activity

**Fig 8:**
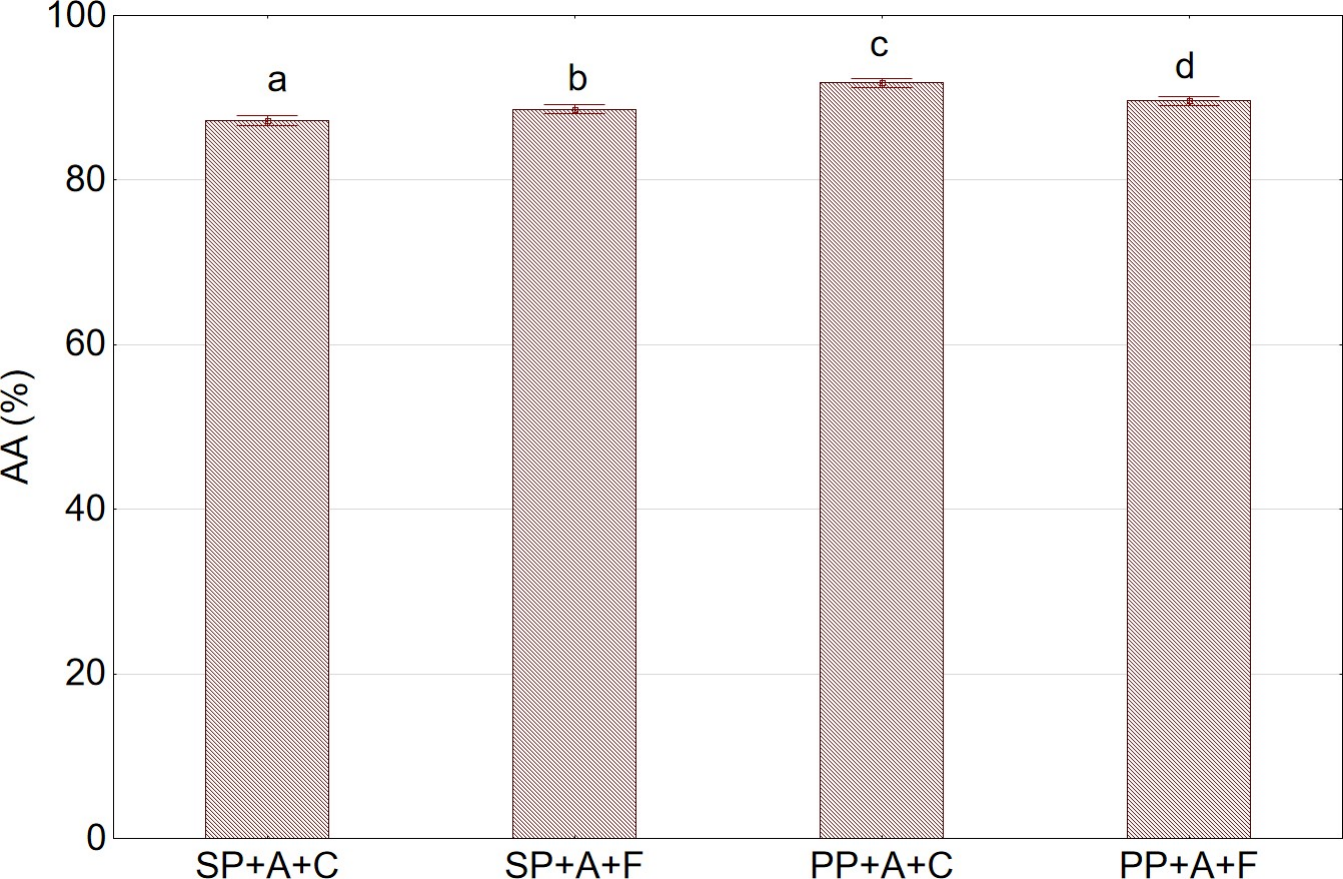
Antioxidant activity (AA) in juice in relation to press type and the method of pretreatment prior to pressing. Abbreviations: SP—twin gear juice extractor; PP—cylindrical piston press; A—nonshredded fruits;; C—sample not subjected to any pretreatment (control); F—sample after pretreatment by freezing and thawing prior to pressing. ^a,b,c^Values in a column marked with the same letter are not statistically significantly different (p>0.05).

Apart from TPC, AA of juices is an equally important predictor of quality. The changes in AA in relation to the press type and the method of pretreatment of aronia fruits are shown in Fig 8.

**Fig 9:**
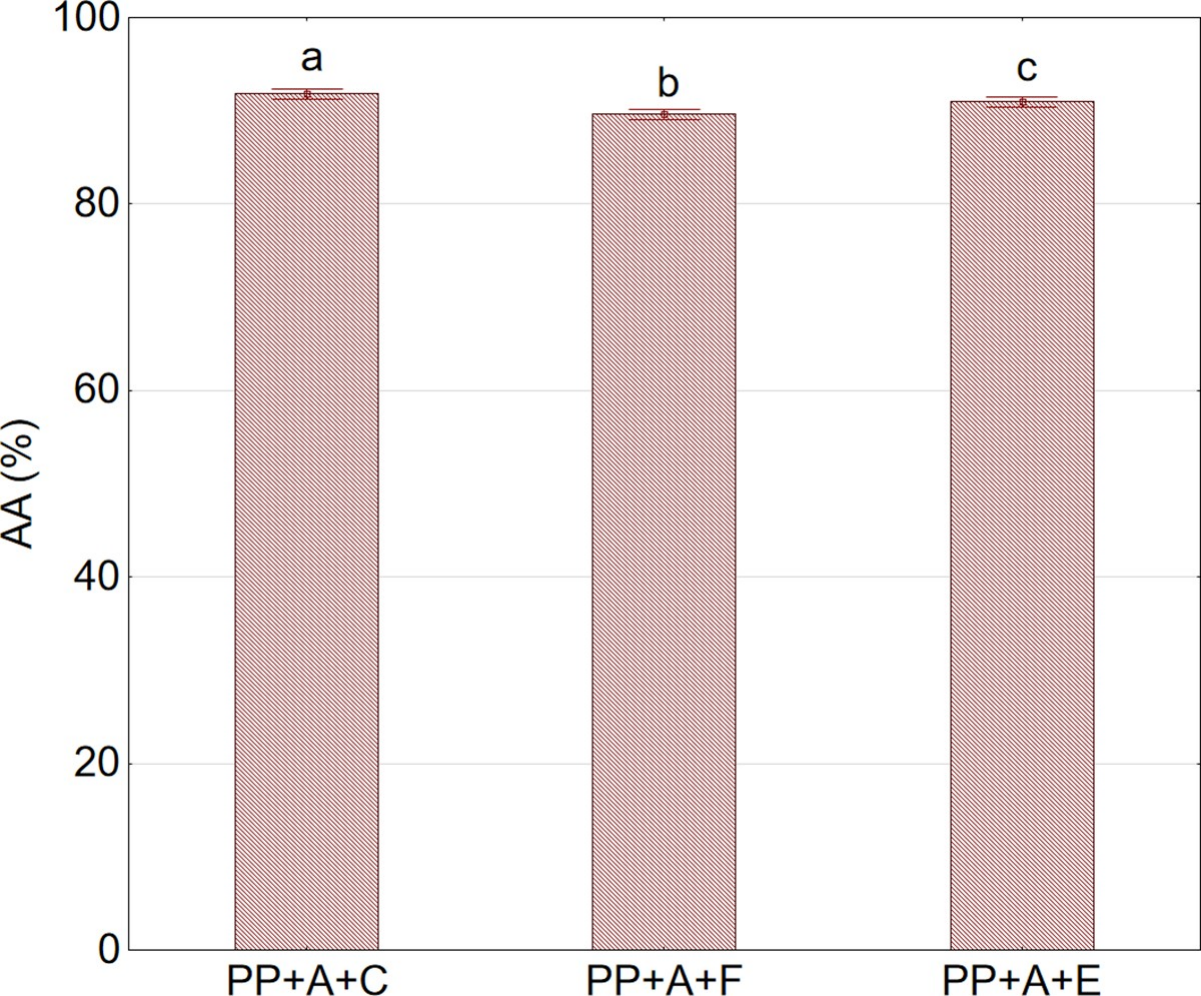
Antioxidant activity (AA) in juice acquired using the piston press in relation to the method of pre-treatment prior to pressing. Abbreviations: PP—cylindrical piston press; A—nonshredded fruits; C—sample not subjected to any pretreatment (control); F—sample after pretreatment by freezing and thawing prior to pressing; E—sample after enzymatic pretreatment. ^a,b,c^Values in a column marked with the same letter are not statistically significantly different (p>0.05).

A slightly higher capacity for free radical quenching was obtained for juice extracted by the piston press than for juice extracted by the twin gear press. Pretreatment consisting of fruit freezing and thawing prior to pressing had an ambiguous effect on the AA of the juices. For the twin gear press, fruit freezing caused a slight increase in AA, while for the piston press, an opposite effect was observed (Fig 8). For juices extracted from whole fruits using the piston press, a statistically insignificant decrease in AA was observed in juice obtained from both frozen fruits and fruits subjected to enzymatic treatment (Fig 9).

Piłat and Zadernowski [32] obtained a lower capacity for free radical quenching in blueberry juices acquired from frozen fruits relative to juices from non-frozen fruits, even though the juices were characterized by a higher TPC. Mirsaeedghazi et al. [33] also observed a decrease in the AA of pomegranate juice stored at approximately −25°C. However, Lohachoompol et al. [38] did not observe any effect of freezing on the AA of blueberry juices, while Polinati et al. [39] did not observe any effect of freezing on the AA of apple juices.

**Fig 10:**
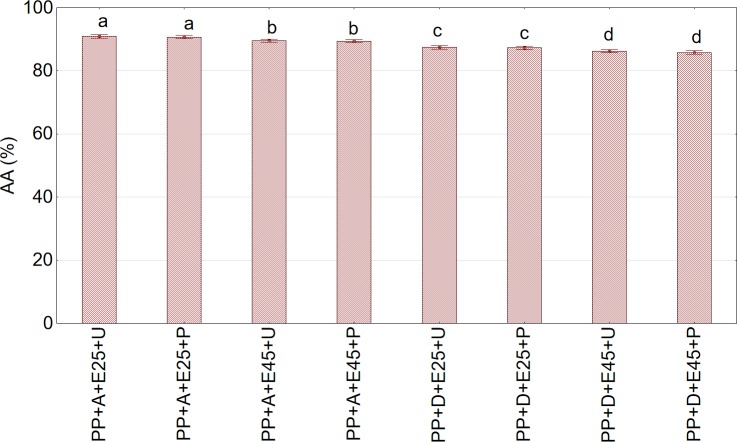
Antioxidant activity (AA) in pasteurized and nonpasteurized juice acquired using the piston press from fruits subjected to enzymatic treatment. Abbreviations: PP—cylindrical piston press; A—nonshredded fruits; D—shredded fruits; E25—sample subjected to enzymatic treatment at 25°C; E45—sample subjected to enzymatic treatment at 45°^o^C; U—nonpasteurized juice; P—pasteurized juice. ^a,b,c^Values in a column marked with the same letter are not statistically significantly different (p>0.05).

Fig 10 presents the effect of variants of enzymatic treatment on AA of aronia juices. The acquired juices were characterized by similar levels of AA (approximately 90%). The highest AA was noted for juices obtained from whole fruits subjected to enzymatic treatment at 25°C, and the lowest AA was noted for juices obtained from shredded fruits subjected to enzymatic treatment at 45°C. Similarly, lower AA after the application of enzymatic treatment was observed by Piłat and Zadernowski [28] during the extraction of juice from lingonberry fruits.

It is worth noting that no statistically significant effect of pasteurization on AA of acquired aronia juices was observed. This result additionally suggests that results regarding the TPC in juices after their pasteurization are of a debatable nature.

## Conclusions

The study shows that pressing efficiency depends on the design of the press and the type of pretreatment. The highest pressing efficiency was obtained using the twin gear juice extractor. For juice obtained by pressing whole fruits using the piston press, the application of pretreatment consisting of fruit freezing and thawing had a significant effect on the pressing efficiency. Enzymatic liquefaction of shredded fruits significantly increased the efficiency of pressing using the piston press. The type of press and the kind of preliminary treatment had an effect on the quality traits of the acquired juices. The highest content of soluble solids was obtained for the pressing of fruits not subjected to any pretreatment using the twin gear press. The application of enzymatic treatment or pretreatment consisting of fruit freezing and thawing prior to pressing caused a decrease in the content of soluble solids in the juice. Irrespective of the method of obtaining juice and the applied treatment, differences in the acidity of the extracted juices were small. The study revealed a distinct effect of the method of juice acquisition on TPC and AA. The highest TPC was obtained in juice extracted using the piston press from shredded fruits subjected to enzymatic treatment at 45°C. A higher TPC was also noted in juice from fruits not subjected to any pretreatment and extracted with the twin gear press. The lowest TPC was found in juice from fruits subjected to pretreatment consisting of fruit freezing and thawing prior to pressing. The capacity of black chokeberry juices for free radical quenching oscillated around the level of approximately 90%. It should also be noted that the methods of juice processing had a little effect on the AA of the juices.

The study showed that the application of suitable processing methods is necessary for the acquisition of a product with desirable quality traits. To satisfy consumer expectations, one should search for such fruit processing technologies through which the presence of biologically active substances in the products will enhance their nutritive value. Taking into account the documented health-promoting effect of components contained in black chokeberry juice, it can be concluded that after the application of appropriate processing methods, the raw material has potential for use in the production of health-promoting foods.

## Supporting information

S1 File Pressing efficiency and juice properties in relation to press type and pretreatment method. (XLSX)
